# A decadal view of biodiversity informatics: challenges and priorities

**DOI:** 10.1186/1472-6785-13-16

**Published:** 2013-04-15

**Authors:** Alex Hardisty, Dave Roberts

**Affiliations:** 1 School of Computer Science and Informatics, Cardiff University, Queens Buildings, 5 The Parade, Cardiff, CF24 3AA, UK; 2 Department of Zoology, The Natural History Museum, Cromwell Road, London, SW7 5BD, UK; 3 Detailed in Appendix 1

**Keywords:** Biodiversity, Informatics, Grand challenge, Decadal vision, Research infrastructure, e-Infrastructure, Data sharing, Systems approaches

## Abstract

Biodiversity informatics plays a central enabling role in the research community's efforts to address scientific conservation and sustainability issues. Great strides have been made in the past decade establishing a framework for sharing data, where taxonomy and systematics has been perceived as the most prominent discipline involved. To some extent this is inevitable, given the use of species names as the pivot around which information is organised. To address the urgent questions around conservation, land-use, environmental change, sustainability, food security and ecosystem services that are facing Governments worldwide, we need to understand how the ecosystem works. So, we need a systems approach to understanding biodiversity that moves significantly beyond taxonomy and species observations. Such an approach needs to look at the whole system to address species interactions, both with their environment and with other species.

It is clear that some barriers to progress are sociological, basically persuading people to use the technological solutions that are already available. This is best addressed by developing more effective systems that deliver immediate benefit to the user, hiding the majority of the technology behind simple user interfaces. An infrastructure should be a space in which activities take place and, as such, should be effectively invisible.

This community consultation paper positions the role of biodiversity informatics, for the next decade, presenting the actions needed to link the various biodiversity infrastructures invisibly and to facilitate understanding that can support both business and policy-makers. The community considers the goal in biodiversity informatics to be full integration of the biodiversity research community, including citizens’ science, through a commonly-shared, sustainable e-infrastructure across all sub-disciplines that reliably serves science and society alike.

## The grand challenge

The grand challenge for biodiversity informatics is to develop an infrastructure to allow the available data to be brought into a coordinated coupled modelling environment^a^ able to address questions relating to our use of the natural environment that captures the ‘*variety, distinctiveness and complexity of all life on Earth*’^b^.

Biodiversity processes are complex and can have a large, long-term impact at the macro-scale, even if they have occurred rapidly at the sub-cellular molecular level e.g., the Phosphate cycle [1,2]. Processes taking place in seconds over scales of nanometres, are crucial to processes that take years and at scales of many hectares and ultimately to planetary processes in geological time. Capturing such inter-dependent processes, across such a breadth of scales, is beyond the capability of current information management and modelling methods. To have an impact on biodiversity conservation, sustainability or our environment, we need to consider all aspects of biodiversity, from genes to ecosystems, in a holistic approach. We need to be able to assess global biodiversity changes and make predictions about ecosystems. We need to be able to integrate different facets of past and present environmental and biodiversity observations and embed them in models with predictive power [3]. We will need to develop new models to address socially urgent questions. Such an approach will take biodiversity science far beyond a collection of taxon names, capturing data about different facets of biodiversity, both by their absolute position and their relative position, together with their observational and temporal context. Most importantly, through biodiversity informatics, biodiversity scientists will be able to understand, to measure and predict how change affects the actual functioning of the ecosystem.

## Recommendations

As well as addressing practitioners with an interest in and knowledge of informatics and how it can be applied to support biodiversity science, our recommendations are intended to inform funders, project managers and institutions whose remit includes at least some aspect of biodiversity science. Our recommendations are intended to establish a background against which decisions can be made when making and evaluating proposals, allocating funds or directing work to build infrastructures. For long-term success, geographically distributed infrastructure involving multiple stakeholders depends on the commitment of those stakeholders to support a vision and to adhere to standards agreed by the community. Stakeholders each have to fund their part in the endeavour to make the whole thing sustainable. Long-term sustainability will be achieved by integrating services provided by key players as part of their core mission.

The first 3 recommendations should apply to all activity in this area. They are necessary to reduce duplication and to enhance collaboration. The intended consequence is to facilitate the creation of new knowledge by synthesis activities using the data and tools thus generated.

1. Open Data [4], should be normal practice and should embody the principles of being accessible, assessable, intelligible and usable [see Context].

2. Data encoding should allow analysis across multiple scales, e.g. from nanometers to planet-wide and from fractions of a second to millions of years, and such encoding schemes need to be developed. Individual data sets will have application over a small fraction of these scales, but the encoding schema needs to facilitate the integration of various data sets in a single analytical structure [see Paragraph 19 et seq.].

3. Infrastructure projects should devote significant resources to market the service they develop, specifically to attract users from outside the project-funded community, and ideally in significant numbers. To make such an investment effective, projects should release their service early and update often, in response to user feedback. [see paragraphs 10 and 31].

While several technologies have already been developed, they are not widely embraced by the community, often due to reasons related to the ‘human factor’. The following 4 recommendations on technological foundations focus on enhancing the usability and better deployment of existing technologies:

4. Build a complete list of currently used taxon names with a statement of their interrelationships (e.g. this is a spelling variation; this is a synonym; etc.). This is a much simpler challenge than building a list of valid names^c^, and an essential pre-requisite [see paragraph 1].

5. Attach a Persistent Identifier (PID) to every resource so that they can be linked to one another. Part of the PID should be a common syntactic structure, such as ‘DOI: …’ so that any instance can be simply found in a free-text search [see paragraph 7].

6. Implement a system of author identifiers so that the individual contributing a resource can be identified. This, in combination with the PID (above), will allow the computation of the impact of any contribution and the provenance of any resource [see paragraph 11].

7. Make use of trusted third-party authentication measures so that users can easily work with multiple resources without having to log into each one separately [see paragraph 12].

The foundational technologies described above all exist to some degree, but need to be integrated. The next steps will require developing new structures by exploiting existing technologies in novel ways.

8. Build a repository for classifications (classification bank) that will allow, in combination with the list of taxonomic names, automatic construction of taxonomies to close gaps in coverage [see paragraph 2].

9. Develop a single portal for currently accepted names - one of the priority requirements for most users [see paragraph 3].

10. Standards and tools are needed to structure data into a linked format by using the potential of vocabularies and ontologies for all biodiversity facets, including: taxonomy, environmental factors, ecosystem functioning and services, and data streams like DNA (up to genomics). [see paragraphs 16 and 17].

11. Mechanisms to evaluate data quality and fitness-for-purpose are required [see paragraphs 20 and 23].

Looking to the future, it is clear that new techniques, such as observatories employing novel sensors are delivering data in unprecedented volumes, especially molecular data, as the Genomic Observatories Network [5,6] has emphasised. This will require development of new technologies, or adaptation of technologies from related fields, new information systems, and platforms offering overviews of detectors and experimental setups for biodiversity research to facilitate exploitation of the opportunities presented.

12. A next-generation infrastructure is needed to manage ever-increasing amounts of observational data [see Paragraph 13, 19, 21 and Appendix 2].

## Preface

*“The Hubbell paper*[7]*made it into BBC*[8]*. It is sad to see where we stand after 20 years. We have done more work, we developed an impressive array of biodiversity informatics, we have tools to capture specimens in our collections and make the data accessible, but the basic we are missing: A strategy to explore the living planet, and even less a strategy to measure the change of species based at least on a basic count of what's out there.”* Donat Agosti, Plazi.

*“When writing my electronic monography (e-monograph) in 2007–9 I wished to link the plant species to other organisms within the ecological food chains / food web. However, I could not even find an e-monograph on birds at the time or have the software programming knowledge to create interspecies relationships between electronic monographs and / or electronic floras. Ultimately I wish to see a ‘virtual life on Earth’ where cross-linking of data can be explored, for example, how shifting species distribution in light of climate change will affect food webs. Consequently the results can be used to drive conservation management and placement on the IUCN Red Data List.”* Fiona Young, University of Reading, UK

The two quotes above illustrate the challenges and associated shortcomings facing biodiversity informatics today. Despite considerable progress, biodiversity science is still reliant on data that is not as fully available, linkable, discoverable and accessible as it should be. Services and tools to process those data are not yet ‘plug and play’. Models of different parts of the overall biodiversity system from the molecular to the planetary are not yet linked across time, space and scales. We are still unable to understand the complex behaviour of the entire system because until now we have reduced it, only taken account of some of its parameters and analysed only parts of it, and just by summing those different parts we cannot understand how the entire system functions.

Biodiversity science is part of the broader drive towards managing our planetary environment, particularly moving to a sustainable pattern of use in the face of a growing human population. Related questions to pose over the next decade include: Will we need an organismal inventory to understand and monitor ecosystem function? Will we be able to monitor functional diversity directly? Can we measure fluxes as a metric of ecosystem health [9]? Will we be able to develop better mechanisms to represent organism interactions, for instance, the microbiome of multicellular organisms [10], viruses in plants or the composition of the rhizosphere? These are comparatively new areas of research not yet represented by a significant body of data or services, but essential for managing our planet in the long-term [3,11,12].

To scale up and understand the whole system, we need new approaches, data types and services. Access to these larger data resources are largely to be found through informatics, but the application of those resources will be made by domain specialists. Our ultimate goal is an understanding of the whole Earth system, so we must retain a broad range of biodiversity monitoring sites, but at the same time we should also focus research effort on key model ecosystems where we can achieve the intensity of outcomes the biomedical research community has with the model organism approach. Only by looking at vast databases that describe the whole of the system will we be able to understand the big picture, find correlations and patterns of activities. Knowing how such patterns and processes of biodiversity change will further help in more targeted experimentation, resulting in new key datasets. Enhancing the biodiversity informatics infrastructure we have today is therefore indispensable.

## Context

The EC Commissioners Máire Geoghegan-Quinn (Research and Innovation), Neelie Kroes (Digital Agenda), and Connie Hedegaard (Climate Action) have emphasised^d^ the crucial nature of infrastructures for achieving their respective political agendas. In particular, Commissioner Neelie Kroes on 11th April 2012 [13], emphasised the importance of open e-Infrastructures, sharing of raw data and results, and collaboration to enable more open science. Open science is the direction that the European Commission (EC) promotes for project proposals under the Horizon 2020 funding initiative, also in accordance with the Nagoya protocol [14], adopted at the 10th meeting of the Parties to the Convention on Biological Diversity [15].

The UK’s Royal Society published a report called ‘Science as an open enterprise’ [4] that highlights the need for a paradigm shift away from traditional practices and mindsets. To quote the report, “… although scientists do routinely exploit the massive data volumes and computing capacity of the digital age, the approach is often redolent of the paper age rather than the digital age”. Key to their vision is the concept of ‘Intelligent Openness’ (Table 1), a standard that Biodiversity Informatics must attain before attempting more complex linkage of services.

**Table 1:** Intelligent openness as defined by the UK’s Royal Society

**Intelligent openness terms**	**Definition**
Accessible	Data must be located in such a manner that it can readily be found and in a form that can be used.
Assessable	In a state in which judgments can be made as to the data or information’s reliability. Data must provide an account of the results of scientific work that is intelligible to those wishing to understand or scrutinise them. Data must therefore be differentiated for different audiences.
Intelligible	Comprehensive for those who wish to scrutinise something. Audiences need to be able to make some judgment or assessment of what is communicated. They will need to judge the nature of the claims made. They should be able to judge the competence and reliability of those making the claims. Assessability also includes the disclosure of attendant factors that might influence public trust.
Useable	In a format where others can use the data or information. Data should be able to be reused, often for different purposes, and therefore will require proper background information and metadata. The usability of data will also depend on those who wish to use them.

Within this context of a more open and transparent future where both scientific results and the data needed for the conduct of science are easily accessible, linked and properly attributed and preserved, we consider the challenges and priorities in a decadal vision for biodiversity informatics^e^ at the European level.

Such a vision is of global interest^f^ and should be the result of a comprehensive strategic roadmapping exercise, like the one recently undertaken in the health informatics domain [16]. It is necessary to engage with the biodiversity community and use mixed methods to elaborate likely future scenarios from which to derive the required strands of future informatics development. On the other hand, we can build on substantial reflective work that already exists.

In “The big questions for biodiversity informatics” [17] Peterson et al. assert that biodiversity informatics currently exists “without major guiding scientific goals that represent intellectual frontiers and challenges”, and fear this gap leaves biodiversity informatics without a framework for effective thinking, resulting in a disjoint set of resources – both data and tools – that cannot be effectively harnessed together yet. They posit a future where biodiversity informatics enables biodiversity science to become a predictive exploration of space, time and form. In “Evolutionary Informatics: Unifying knowledge about the diversity of life” [18], Parr et al. propose the grand goal to "Link together evolutionary data across the great Tree of Life by developing analytical tools and proper documentation and then use this framework to conduct comparative analyses, studies of evolutionary process and biodiversity analyses". Five challenges to realising that goal are also discussed.

In this white paper we must establish a stronger focus and a direction to guide the development of biodiversity informatics in Europe over the next decade whilst at the same time allow for serendipity. Clearly, the rate of change in the technology environment around us is dramatic: tools like Facebook, Twitter, Foursquare, Google (Earth/Scholar/…), Mendeley and Dropbox have penetrated our working lives and techniques like MapReduce [19] impact greatly our ability to manipulate and analyse massively large datasets. Smartphones, digital cameras, GPS positioning and progress in geospatial analysis offer possibilities for ‘apps’ and techniques that were hardly imagined just a few years ago. Workflows as a tool for in-silico processing of data and the concept of Virtual Laboratories where scientists carry out digital experiments, hardly known few years ago, offer today enormous opportunities in virtually reproducing our environment. Similarly in genomics, the rapidly decreasing costs of sequencing technologies combined with the emergence of increasingly sophisticated alignment and inferencing algorithms is leading to huge increases in our knowledge of life as a system.

This ‘disruptive’ innovation trend will continue with ‘cloud’, ‘big data’, ‘linked data’ and ‘open access’ leading to new ideas, products and services. The biodiversity informatics community is adapting to the increasing rate of change by adopting dynamic solutions freed from rigid technologies that may be obsolete tomorrow. We need to demonstrate how we are joining up. Collectively we need to see the big picture, understand the jigsaw of challenges and decode all the complexity that exists within populations and species.

To put this in context, two recent initiatives have, respectively, examined the challenges facing biodiversity science research in Europe, and espoused the architectural principles of the Biodiversity Observation Network (GEO BON) within the Global Earth Observation System of Systems (GEOSS) initiative. The first initiative led to the LERU report [20] that discusses 18 building blocks for the biodiversity research agenda, necessary to implement the EU’s 2050 vision for biodiversity and ecosystem services^g^, and to reach the EU’s 2020 targets^h^ for halting biodiversity loss. Principally, the report points to the need for a common e-Science infrastructure for biodiversity research (sub-clause 13). Several of its key recommendations involve informatics playing a substantial, enabling role. With their clause numbers from the LERU report in brackets, these recommendations are:

• Investing in a European infrastructure for biodiversity data and research (sub-clause 32)

• Investing strongly in enhancing fundamental knowledge on biodiversity drivers and threats (sub-clause 33)

• Supporting effective translation of scientific knowledge into biodiversity practice (sub-clause 34)

• Supporting multidisciplinary collaborative networks (sub-clause 41)

• Supporting the science-policy interface in biodiversity protection, and in particular supporting the needs of the Intergovernmental Panel on Biodiversity and Ecosystem Services (IPBES) (sub-clause 42)

• Delivering education and awareness (sub-clause 43).

The second initiative is GEO BON [21-23] that, building on existing networks and initiatives, proposes "an informatics network in support of the efficient and effective collection, management, sharing, and analysis of data on the status and trends of the world’s biodiversity, covering variation in composition, structure and function at ecosystem, species and genetic levels and spanning terrestrial, freshwater, coastal, and open ocean marine domains".

GEO BON fits in the broader conceptual framework of GEOSS [24] to deliver a decentralised and distributed informatics infrastructure. The GEO BON system will have a Service Oriented Architecture (SOA) and will be built largely from contributing systems that have their genesis at regional, national or sub-national scales. At the European level, the planned ESFRI LifeWatch research infrastructure [25], along with EBONE [26] and EU BON [27] projects, eventually forms the European contribution to realising GEO BON.

Key among the technical objectives of GEO BON is the need to promote the use of multidisciplinary interoperability standards, and to define and update interoperability solutions – applying the System of Systems approach promoted by GEOSS. GEO BON will also help to promote data publication principles in support of full and open availability of data and information, recognising relevant international instruments and national policies and legislation. One of the main tasks for GEO BON contributors is thus to identify the main contributing components, list the services they provide and also the standards or special interoperability solutions they use. Central to the success of GEO BON is increasing cooperation among the standards organisations with interests in the biodiversity science domain, notably: the Genomics Standards Consortium (GSC) for standards at the genomic / genetic level; TDWG for biodiversity information standards at the organism level; and the Long-Term Ecological Research network (LTER) for standards concerned with populations and ecosystems. Better cooperation leads to better coherence among standards and better interoperability.

It is clear, then, that the landscape of biodiversity informatics is already complicated, but is understood. Moving forward must take account of, and build upon, what has already been achieved.

## The taxonomic impediment

The term Taxonomic Impediment [28] was coined by IUBS/Diversitas to refer to the gaps in our taxonomic knowledge, the shortage of taxonomic expertise and the impacts that these have on the progress of biodiversity science. In this context, taxonomy is the knowledge that allows the actors in a process to be identified and for inference to be drawn from the presence of a particular organism. Taxonomic services refer to the means of delivering that knowledge and present three basic problems:

• Taxonomic services rely on highly educated personnel and hence are very expensive;

• The data delivered by traditional taxonomic services have a limited application potential, largely because species identification is expensive and therefore typically carried out with a limited spatio-temporal and taxonomic scope, unsuited to address ecological questions at larger spatio-temporal scales or more complex patterns;

• Taxonomic expertise is shifting away from traditional practices of producing morphological descriptions and identification keys towards phylogenetic, especially molecular, studies.

The Taxonomic Impediment is, in part, a reflection of the need to use these expensive taxonomic services for all studies in the natural world. Alternative approaches that could address some biodiversity-related problems could help to relieve the currently perceived bottleneck and allow taxonomists to focus on those groups where their skill delivers greatest return. Necessary tools include semi-automated image-based species identification services based on techniques such as those described in [29] and citizen reporting systems such as the Swedish Species Gateway [30]. Enhancing taxonomic services with DNA-based identification tools (e.g. the DNA Barcode of Life standard [31]) for example will not only improve the quality of identifications (objectivity, data interoperability), but will also deliver high-throughput approaches for environmental monitoring, species intense ecosystem research (e.g. Moorea Biocode Project [32]), and better ecosystem-based management.

Biodiversity informatics can help by liberating the taxonomic scientist from the clerical labour of locating comparative materials, both specimens and literature^i^[33].

A more radical way to overcome the taxonomic impediment might be to use biodiversity informatics without traditional taxonomy. Molecular studies can generate characteristic sequences, to identify organisms, or more radically still, identify particular enzymes central to the process of interest. It could be argued that the current thinking of species name and location is paper-based and not embracing the informatics potential. Research projects exploring such innovative approaches should be encouraged. In particular genomic observatories are in a position routinely to sequence DNA and link this foundational layer of biodiversity to its biological, ecological, environmental and social context.

## Changing the landscape - a decadal vision

The key component needed to develop biodiversity informatics further is effective integration of the available resources, to ensure that the practice of publishing biodiversity information becomes widely adopted in the scientific community and leads to scientific synthesis. Synthesis is increasingly recognised as an essential component of the scientific endeavour. Scientific synthesis refers to the integration of diverse research in order to increase the generality and applicability of the results. At its core, synthesis is about blending disparate information and knowledge in ways that yield novel insights or explanations [34]. Synthesis occurs both within and across disciplines and the implementation of an effective biodiversity informatics infrastructure would greatly enhance this type of activity. Such an enhanced integration of all related information, including raw data, processed data, algorithms (code, workflows) and publications can be achieved through the implementation of an effective biodiversity informatics infrastructure: a shared and maintained multi-purpose network of computationally-based processing services sitting on top of an open (published, registered and linked) data domain. Together, these deliver a stable, broad portfolio of biodiversity information and analytical services that can be used by user communities to investigate problems of interest.

The vision is to develop the concept of 'services' delivering either data or analysis of information using a small set of interchange standards. New services can be introduced into such an environment and be generally accessible without special effort. This vision implies a number of significant details, which are elaborated in more detail in the remainder of this white paper.

## Realising the vision

Effective realisation of the decadal vision relies on achieving a balance of top-down and bottom-up approaches by making appropriate funding decisions. Top-down approaches include thinking and acting at the European level, encouraging community adoption of standards within the EU (part of a worldwide effort in which the EU is a key player), setting direction and goals through targeted funding calls, workshops and meetings. Bottom-up approaches derive from the motivation of individuals, their ideas and enthusiasms and their need to solve specific problems. Both approaches together recognise the role that individuals and groups have to play in the decadal vision by encouraging islands of infrastructure to emerge, grow organically and fuse with one another over time.

## Leveraging existing projects

Numerous biodiversity informatics projects have been funded in Europe by, amongst others, the Framework Programmes. Globally, there are already more than 650 projects known [35]. Examples from Europe include the networks of excellence (ALTER-Net, LTER-Europe, EDIT/PESI, MarBEF/Mars, EuroMarine etc.) and other projects such as 4D4Life/i4Life, agINFRA, Aquamaps, iMarine, BioFresh, BioVeL, ENVRI, EU-BON, EUBrazilOpenBio, Fauna Iberica, MicroB3, OpenUp!, pro-iBiosphere and ViBRANT among others^j^. Many of these projects directly address the challenges of deploying e-Infrastructure for biodiversity science^k^. They seem to share similar characteristics, such as scientific field, integration and interoperation of resources, open access, service orientation, e-Infrastructure and e-Science virtual environments. They differ substantially, however, in their architectures and technological approaches. These largely technical differences illustrate a larger problem: the lack of a common understanding about how best to deploy e-Infrastructures for biodiversity and ecosystem research. None of the projects can solve the problem alone nor hope to provide all the functionalities that will be needed in the future. Working on non-converging agendas, understandable given the imperative to push boundaries for innovation and academic advancement, does not lead to a coherent infrastructure with all necessary capabilities and capacities to support scientific research. There are overlaps, dead-ends and often, complete lack of mainstream industrial involvement. It is for such reasons that community consensus around a decadal vision, combined with effective selection of projects to be funded and their subsequent interactions and management within a coherent programme, is so important.

The decadal vision provides the means by which the complementary aspects of multiple projects can be combined in a common roadmap forward. Achieving this requires an increased awareness from all projects of the architectural approaches and construction steps to be adopted. Multiple projects contributing to that infrastructure need to get aligned because no single project can solve all problems alone. Separate projects need to achieve greater coherence and coordination to maximise the benefit from substantial investments of the past, present and future.

Within the Horizon 2020 framework it is therefore required to develop an effective and continuing coordination, dissemination, education and training capability providing and re-distributing help, technical guidance and examples of best practice. This capability will inform individuals and groups about the top-down strategies, the priorities and progress made, leading towards greater community understanding of the overall vision.

Project proposals developed bottom-up for Horizon 2020 funding should fit under the umbrella of the community’s decadal vision. They should leverage completed and existing funded projects to gain the maximum benefit for the future biodiversity infrastructure. Proposals should explain how they have taken earlier and current project results into account and demonstrate that they are building on them rather than offering incompatible alternatives. Re-inventing the same (or different) solutions is not cost-effective. Letters of support from other projects should be used to demonstrate that community-wide discussion and acceptance of proposals has taken place prior to submission for funding. Project proposals should show clearly where and how they contribute towards the decadal vision. They should devote a significant portion of their resources to networking with other projects, to demonstrating compatibility and added-value as a key performance indicator at an early stage, and to marketing the services, technologies and approaches being developed to potential users.

## Section 1: the fundamental backbone (getting the basics right)

### Why are names important?

1. Until the recent application of molecular technologies to biodiversity studies, almost all information has been labelled with scientific names. Names have a special significance to link information elements and as such, it is important to use them knowingly and to build tools that work with names [36]. As they reflect concepts that change between individuals and over time, names may refer to many different concepts, making them equivocal identifiers. In addition, information is often available only in local databases. The challenge is to find it, harmonise the way it is accessed and make it available in computer-readable formats. Nomenclature, taxonomy, taxa and their biology together constitute a large challenge requiring novel infrastructure and change of usual practices by stakeholders. Numerous initiatives exist to deal with these aspects but progress will require a common agenda to bring about a virtual infrastructure that will reduce the apparent diversity of web resources without reducing the diversity of services required by a diverse user community. While content about taxon names must be assembled by nomenclaturalists, taxonomists and managers of biodiversity information, there is an urgent need for vision-driven architectural and engineering solutions. The GNA’s (Global Name Architecture) [37] current priority is on name-strings (Global Names Index GNI [38]) and name-usage instances^l^ (Global Names Usage Bank GNUB [39]). The latter does not yet exist but will provide the essential semantic relationships (cross-links) at the nomenclatural level. This focus is entirely appropriate because universal coverage is tractable in the short to medium term. The resolution of names to concepts (see paragraph 3, below) is far more difficult and is likely to be intractable for universal coverage.

### How are names organised?

2. A long unorganised list of names is not particularly helpful. Since Linnaeus biologists have used latinised binomial names where the first part (the genus) is shared by a group of similar organisms and the second part, the epithet, differentiates between members of the group (e.g. oak trees belong to the genus *Quercus* that contains around 600 species). A similar hierarchical classification is followed for genera that are grouped into families, families into orders, orders into classes and classes into phyla. As science advances, however, these relationships change with greater understanding. While it is possible to build hierarchies from instances of name-strings, it is inefficient. The solution required includes a classification bank combined with a name list (see Paragraph 1) to produce a taxonomic hierarchy automatically for groups that have not recently received taxonomic attention.

### Which is the right name?

3. The Species2000 / ITIS Catalogue of Life [40] is a global taxonomic reference system drawing on content from more than 100 sources. It provides a composite expert view on taxonomic information, providing an authoritative but mutable framework. Names within CoL represent concepts, but there is no link to the concepts themselves and therefore an identification cannot be unequivocally verified. Other classifications with names, such as NCBI taxonomy [41] or the WoRMS systems [42] can also be used as organisational frameworks. Yet each serves its own audiences, revealing the need for multiple systems that are however interoperable. Initiatives such as the Global Names Architecture (GNA) [37] promote the development of an infrastructure capable of linking available information about biological names. iPlant’s Taxonomic Name Resolution Service, TNRS 3.0 [43] corrects and standardises plant scientific names against particular taxonomies. ZooBank [44] is a new initiative to move the process by which new names become recognised into the digital age. Tools for alignment and cross-mapping of taxonomies can only be partially automated, since the domain knowledge held by taxonomists is very difficult fully to codify. Some projects such as i4Life [45] have developed tools that exploit characteristics of biological nomenclature to detect relationships between taxonomies, providing a useful "first draft" cross-map. Nevertheless to be authoritative, future environments must link nomenclators (like ZooBank, IPNI and Index Fungorum, Mycobank), taxonomic compendia (such as CoL and WoRMS), other classifications (a classification bank, perhaps), literature sources (describing species, their attributes, distributions, and common names), and phylogenies, covering the whole spectrum of biodiversity complexity. The taxon name is an access key, but it is essential that it can be linked to other resources, such as descriptions, traits and habitat. Ultimately names are the bridge to the accumulated information built over the past 300 years and trapped in the paper world.

### What is the name of that organism?

4. The practical identification of an organism relies on the construction of a circumscription of the taxon to be identified, which in turn requires the examination of a range of specimens agreed to belong to the taxon. Before the digital era, the only ways to identify the name of an organism were to use a paper identification key or to consult an expert. New identification techniques have emerged to get to the name of an organism, including matrix keys and ‘smart keys’ that use the locality and time of the year to reduce the number of identification choices. These identification techniques however are labour-intensive and depend on experts to create the necessary circumscriptions, keys and the link to the list of accepted names. Automated identification techniques, like image recognition or *in situ* DNA analysis, are not yet sufficiently developed to be used routinely and reliably for most organisms. Identification keys always cover a small part of biodiversity and may also be difficult to discover. Developing morphological keys to all organisms is not achievable because no global organisation can establish a central repository, or even coordinate, prioritise and fund the creation of keys. The major priority therefore is to make the necessary descriptive data with their associated range and habitat information freely available. Services can then be created, for instance as 'apps' for the mobile phone market.

### Can biodiversity studies be done without names?

5. Almost all of our accumulated knowledge about biodiversity has been gathered and organised using species names. According to a recent exhaustive review by Costello [33], taxonomists think that about 1.5 million living species have been described, but lacking a single authoritative list of names, this is only an approximation and many species may be invalid [46]. The number of species left to be discovered are substantial [47] and, given that current taxonomy is the product of more than 250 years of effort, it is unrealistic to have a complete catalogue if we adhere to currently accepted methods [33,48]. Solutions include modern molecular techniques, such as DNA barcoding and massively parallel high throughput sequencing, effective at revealing much of the undescribed diversity. Such systems based on environmental genomics (‘metagenomics’) are already well established in microbial ecology where DNA sequences act as tags identifying organisms in the ecosystem. These techniques, being inherently destructive, cannot yield a traditional specimen, so cannot be used to name a new species, but they are promising in assessing ecosystem biodiversity without the requirement to name every species present. Challenges in the deployment of such techniques include:

• Ensuring that the data, information and knowledge emerging from this new paradigm become integrated with traditional taxonomy so that we continue to benefit from the efforts of taxonomists over the previous 250 years;

• Curating the species’ names that have been attributed to sequences in databases of the International Nucleotide Sequence Database Collaboration (INSDC);

• Devising a framework to integrate the specimen-centric observations encoded by the Darwin Core standard with the environmental and ecological context of metagenomics;

• Putting these different layers of information together so as to identify the response of the ecosystem to environmental change;

• Being able to access covariate data, for example concurrent observable chemical, biological and other environmental variables from the target ecosystem, especially for environmental metagenomic studies.

Ecological research will largely benefit from such new approaches classifying and understanding genomic biodiversity based on functions, their evolution and distribution.

### Biodiversity data beyond names

6. Names are an access key to biodiversity information, including information on the occurrence of species in time and space. Ultimately we need to be able to integrate biodiversity information indexed by names with information on:

• Functional diversity;

• Diversity at various levels of organisation: genes, organisms, ecosystems, landscapes;

• Relationships between facets of biodiversity and ecosystem functioning and services;

• Those variables and data that describe the physical environment;

• Fluxes through environments, such as phosphorus into and out of a system.

Crucial to this endeavour will be our ability to devise methods to link such data and to define data standards to make such linkage straightforward (see Paragraph 15 et seq. below).

### To link resources we need identifiers

7. Biodiversity science needs to adopt a system of persistent and universally unique identifiers (PIDs or UUIDs) that will allow resources to be located and linked. An identifier can be attached to a resource of any kind, including data (e.g. specimens in a collection), taxonomic concepts, genetic sequences (e.g. INSDC accession numbers), publications (e.g. DOIs), or data sets and services, (e.g. workflows, computational services or computer code). Identifiers must be stable and unique but they should also:

• conform to some widely-used syntactic definition;

• their initial part should be consistent (e.g. http:// or DOI:), so that they can be recovered in a free-text search;

• ideally, be resolvable (resolution = where to find a particular resource);

• be archived together with the resource in a sustainable manner, ideally in multiple locations (if the GUID is not resolvable, the resource can be found by searching for the GUID).

PIDs do not protect against duplication, i.e. a single resource carrying multiple PIDs, but if they were used, then resources could be linked, so that discovery of any item in a chain of connections would permit the discovery of all the rest of the resources as well as allow for consequent credit allocation (see paragraph 11). There is no technical challenge in the use of PIDs. DOIs, for example, are now familiar in many publications and although the DataCite [49] initiative has significantly reduced both the cost and complexity of using DOIs, their direct application by the biodiversity informatics community remains rare. The community, then, seems reluctant to use PIDs. It is not clear whether this is due to reluctance to change current working practice or whether it is due to lack of suitable tools - either for linking or for following links. Note that a well-known resolver, comparable to CrossRef for DOIs, is only necessary for resolvability (point and click). PIDs, by their unique nature can be discovered with standard search tools such as Google. More elaborate linkage mechanisms are possible, and could deliver much greater benefit, but introducing the community to simple linkage is a challenge, so Linked Data [50] is considered a “next step” (Section 2) rather than a foundational technology in this white paper.

### Centralised or networked services?

8. Networked services refers to the use of a resource directly over the web, so that one website may call another site for information necessary to carry out its function. Centralised services, on the other hand, concentrate all the associated resources at a single site. Although networked services are desirable to maintain consistency and to focus resources for maintenance (i.e. an authoritative master copy), in practice they are often unable to deliver the speed of response necessary for usability and creating a local copy (a snapshot of a dynamic resource) or developing an independent resource is often the only realistic solution available. Local copies are often the only practical solution for computation but there is no mechanism by which alterations to the primary resource can be effectively propagated to all copies. This inevitably leads to differences between copies. Copies should, therefore, be used over a short timeframe and, if necessary, refreshed. A feedback mechanism is required so that a data user can report an inconsistency to the data owner and, where a correction is suggested, can be easily incorporated into the original dataset. Automated workflows crucially require Web services but working with large datasets in networked services poses technical challenges in the ability to move large volumes of data, the provision of suitable search facilities that minimise the number of host-client interactions, and the bandwidth necessary to keep response times short. Centralised services, such as VertNet [51] assemble large collections of data in a common structure, submitted by individual data creators. The benefit that this brings is economy of scale and the ability to tune the performance of the system. In the context of data cleaning, for example, having the data centrally makes it much easier to compare across data sets and discover inconsistencies. The gain of economy of scale is very important since once a given type of error is identified, rules can be applied for cleaning across the whole data set, therefore avoiding overloading remote services. Once established, though, it is difficult to change the structure and change the purpose, but for large-scale data generation systems, logically centralised services offer significant advantages. One drawback, however, now receiving attention in the genomics and other fields is the issue of time taken to move large datasets to where the resources for computational analysis and modelling are located. Strategies are presently being considered for how to move computation to the data.

### How to balance professional and non-professional contributions

9. Engagement of the biodiversity expert community is undoubtedly a key factor in advancing knowledge. Citizen science projects have been remarkably successful in advancing scientific knowledge by providing data primarily on species occurrence and distribution around the world [52]. These engage the public in the collection and analysis of data sets from multiple habitats and can span long periods of time. The big scientific issue tackled by these large data sets is how biodiversity varies through space and time, including biodiversity loss and detection of trends, such as shifting distribution boundaries. Citizen science projects represent a massive effort spent on biodiversity monitoring that could not otherwise be covered by the professional community alone without huge sustained financial investment. The primary challenge for the biodiversity informatics community is to develop a framework to address the currently multiple, cross-cutting requirements of citizen science projects, such as:

• Covering all steps in the development and implementation model of such projects, from the choice of scientific question to the evaluation of the outcomes;

• Automating validation (quality assurance, quality control and data cleansing processing) and annotation of the data produced [53];

• Developing incentives to encourage participation in processing, analysis and use of data;

• Developing virtual research and teaching environment(s) for citizen scientists, to develop their skills to answer basic scientific questions;

• Improving systems for automated image recognition based on existing technologies (e.g. TinEye Reverse Image Search [54]) to harvest the vast repositories of amateur naturalists' photos;

• Promoting best practices by disseminating successful examples of actions on nature conservation;

• Ensuring continuous economic viability for the services through the linking of such citizen science projects with the relevant economic sector’s stakeholders [55].

### Engagement of Users

10. A great deal of high-quality software, services and resources have been created over the past decade, but much remains underused, even within the biodiversity informatics community itself. Many projects have relied on traditional routes to publicise their products, primarily through academic publications. It would be undesirable to impose standard applications or resources upon the community. Better to allow users to decide, to select which products best match their requirements. Projects should invest significant resource into marketing their products, engaging with real users and refining the product from user feedback, following the dictum of "release early and release often". Such marketing need not absorb a significant fraction of a project's budget but should be a clear strategy and an integral part of project management.

### Who's who?

11. Traditionally, experts have published their observations and conclusions in peer-reviewed paper publications, a tradition that has been effectively transferred to the digital age through e-publication. The tradition has several consequences. First, it has created a system of citations by which individuals are assessed for career development. Second, the cost of print-on-paper has driven data presentation to a compact, often summarised, format. Third, the financial interests of the publisher have restricted the availability of the data for re-use. Two aspects of the citation mechanism are important:

• Provenance, meaning that a data user can easily discover who generated the data, which can attach a level of reliability to the data; and

• Impact, by providing a hyperlink that allows a user to see where a particular data set has been used, both how often and for what, which could easily be incorporated into the managerial assessment of an individual’s career.

Modern digital publication could effectively remove the typographical restrictions [4] making data more easily available for re-use. Some publishers, e.g. Pensoft [56], are already introducing publication in parallel formats (paper, PDF, HTML, XML). The new paradigm is about evolving new methods to identify contributors and users consistently, where identification can be carried from one environment to another, including the popular social networking environments like Facebook and Twitter. Approaches to this are being developed in the ORCID consortium [57] and VIAF [58], designed for those who publish scientific articles (scholarly authors), but also need to include other users, such as compilers of reports and assessments. This transition to reusable data identifiably associated with an individual or group of individuals is a common call within the Open Science movement, relevant for all scientific disciplines. Note that the US National Science Foundation now requires applicants for funding to list his or her research “products” rather than “publications”, implicitly recognising the value of contributions beyond paper publication [59].

### User identification

12. Open access data and services allow users to remain anonymous for some level of access. Some forms of interaction however, such as posting comments, corrections and some types of services, such as download, often require that users identify themselves. Social media tools like Facebook, Google and Twitter offer common 3rd-party authentication mechanisms that can be used for access control. This has two main advantages: first, it makes every resource easy to access; and second, it is a stronger security check compared to inventing a username and password for each site visited. Nevertheless, some resources will require a stronger form of authentication, for instance where payment is required. As a general principle, access to biodiversity data should normally be unrestricted except where it is essential to protect, for example, location data for rare bird nesting sites.

### How do we ensure the right metadata are created at the point of data generation?

13. The scientific process requires the collection of observations from which hypotheses can be formed and, when necessary, more data to be collected to test them. Adding metadata represents an overhead on current practice but it is essential if data are to be discoverable and re-usable. Metadata are the key to discoverability and provide the context for linking resources. To improve current practices there is an urgent need for i) community agreement on metadata standards for specific purposes and ii) mechanisms to collect and append the necessary metadata, automatically whenever possible, such as the design of workflows that make use of standard services to create data-recording templates. In the short term, the extra effort of metadata production will have to be borne by the data producer, especially in the context of data journals, but tools to automate the production of metadata are conceivable, essentially eliminating the burden of production. A move to Linked Open Data is expected to obviate the need for enlarged metadata by making data more easily discoverable through concept linkage (see paragraph 15 et seq. below on Linked Data).

### Sustaining the physical infrastructure

14. Appropriate biodiversity informatics tools will generate greater impact than is currently possible from the physical infrastructure of natural history collections, mesocosms, other experimental facilities, long-term ecological monitoring sites and genomics facilities, through much greater digital and on-line access to the facilities than is physically practical. This will enhance the sustainability of the infrastructure, since a large user base is critical for political sustainability.

## Section 2: the next steps

### Data sharing

15. Two relatively large surveys were conducted to understand how data are treated by scientists across different disciplines: by the PARSE.Insight project [60], with 1202 respondents, and by Science Magazine [61], with 1700 respondents, both with multidisciplinary international responses. From what researchers say about where they store and manage data, it can be deduced that data are not often shared openly. The results show that across all disciplines only between 6-8% of the researchers deposit datasets in an external archive of the discipline/research domain. The most common environment for storing, managing and re-using data is the lab and/or individual working environment, down to PCs and portable storage carriers. The category “server” is probably best understood as a file server of the research organisation behind a firewall and with restricted access for defined groups of registered users. According to Science Magazine, most of the respondents (80.3%) said that they do not have sufficient funding available for data curation. Other reports [4,62-65] share more insight into data sharing practices by research area and highlight the importance of data sharing becoming normal practice.

### Why do we need vocabularies and ontologies?

16. Common vocabularies are the foundation for both human and machine communication (e.g. in data sharing, in automated workflows, data integration and analysis). By agreeing on a set of concepts and their definitions within a domain, a community of practice can share data and information unambiguously. Data integration and analysis critically requires semantic consistency as well as syntactic standardisation, the former being more challenging to achieve than the latter. Initially communities will accept a small controlled vocabulary - terms supported by human-readable text definitions. As terms are rarely independent of one another, the vocabulary list evolves into a thesaurus and, as formal relationships between terms are agreed, an ontology [66]. There are lessons to be learnt by looking elsewhere, for example, Google’s "Knowledge Graph" [67], the Unified Medical Language System (UMLS), medical informatics) [68], AGROVOC (agriculture) [69] and OBO (plant and animal phenotypes) stable of ontologies [70]. AGROVOC covers many of the terms relevant to biodiversity and is modular enough to be extended. There are other ontologies useful for capturing biodiversity data, such as the environment ontology, EnvO [71], and the more general DAML [72]. There is a pressing need for ontologies that span multiple communities, implying domains, and at present, such over-arching technologies do not seem to exist. Individual community ontologies tend to isolate communities rather than enable more open sharing, but community ontologies are with us now and need to be integrated. Some systems, such as UMLS are not structured to support reasoning or subsumption, so are not necessarily a good model for further development. Nevertheless, establishment of community standard terminologies and ontologies presents problems that are familiar to other communities, such as human genetics and model organism functional genomics, and some of these lessons have already been learned:

• Terminologies / ontologies need to be owned by the community but their maintenance is an ongoing requirement which requires stable funding and a degree of community coordination and interaction;

• tools that biologists find intuitive need to be developed for both data coding and analysis, making the process efficient and effectively invisible;

• ongoing terminology and syntax development need expert construction and are not just problems of computer science;

• a significant problem exists in the communication of changes in those lists to sites that consume the data and a central catalogue / source is required, such as currently provided by OBO or the NCBO (National Centre for Biomedical Ontology);

• mapping of data coded by legacy terminologies and integration of data coded by different species-specific ontologies are problems already addressed by some communities.

There is potential in semantic interoperability for biodiversity data, but this requires quite basic research and IT development to enter new paradigms supporting open semantic approaches. The provision of a strategy for transferring “legacy” data models into semantic-aware technologies is clearly desirable because existing data models are often accurate, comprehensive and represent a great deal of effort from the scientific community. We need a pragmatic strategy for mobilising this knowledge. Such mobilisation may also assist in achieving broad user acceptance, a greater problem than are the associated technical issues. Developing and applying vocabularies is clearly hard and requires the existence of persistent identifiers (paragraph 7 above) to be effective. It will require organisation and cooperation, or to put it simply, it takes goodwill but also cash.

### How would knowledge organising systems help?

17. The term ‘knowledge organisation system’ (KOS) covers any system for organising information, ranging from the traditional library subject headings to newer approaches like semantic networks and ontologies. Recognising the need for a standards architecture to provide basic interoperability across open systems, the TDWG Technical Roadmaps in 2006, 2007 and 2008 all identified community-supported vocabularies and ontologies, expressing shared semantics of data, as one of three required components; the other two are common exchange protocols and use of persistent identifiers for the data. The TDWG Darwin Core [73] glossary of terms is amongst the most widely deployed biodiversity vocabularies and both its management and relation to the TDWG Ontologies can be used as a model for other vocabularies. The GBIF LSID-GUID Task Group [74] highlighted the need for GBIF to identify sustainable support mechanisms for essential shared vocabularies and commissioned White Paper Recommendations on the use of Knowledge Organisation Systems. GBIF [75] separated the need for ontology management from the lifecycle management of flat vocabularies in such tools as BioPortal. The development, management and governance of such vocabularies remain a challenge for the biodiversity community. As concluded in paragraph 16 and discussed in section 4, the core technologies are available and well understood, but uptake by the community is not ideal. The challenge is to develop and deploy tools within the overall biodiversity informatics infrastructure that make the implementation of knowledge organisation systems effectively invisible. GBIF’s Integrated Publishing Toolkit [76] is one example of a step in this direction. Put simply, what would it take to make knowledge organisation work effectively and what would it achieve if it did?

### How easy is it to integrate data?

18. Biodiversity informatics is inherently a global initiative. With a multitude of organisations from different countries publishing biodiversity data, our foremost challenge is to make the diverse and distributed participating systems interoperable in order to support discovery and access to those data. A common exchange technology, e.g. XML or JSON, may allow the syntactic exchange of data blocks, but both systems also need to understand the semantics of the data being delivered to process it meaningfully. If the data do not share a common reference model, then the exchange requires some brokering or other semantic processing (using tools described in paragraphs 3, 7, 12 and 16 above). For instance, the widely used standard Darwin Core is predicated on the occurrence (either a physical specimen or an observational record) as the unit of information, so is of limited value in the context of metagenomics for example, that may contain information about environmental function without mention of a named taxonomic entity, or information about communities of taxa. It is crucial that future efforts in this area take account of major global initiatives, especially GEO BON, GBIF and Genomics Standards Consortium, as well as novel approaches in eco-informatics, but it is likely that the data models used in these initiatives will also need to be extended [77]. Existing data must either be transformed in a semantically-aware manner to conform to such standards, or software that is aware of the semantic heterogeneity must work with multiple standards.

### Beyond sharing and Re-use: the problem of scale

19. It should be straightforward to assemble a dataset on biodiversity and reach conclusions by linking available information. To understand and model processes, such as the phosphate cycle, requires information at the molecular level over seconds (solubility, diffusion and uptake), kilometre level over years (transportation and availability) and planetary level over geological time (mineral formation, extractability). The integration of all these data resources is necessary to model the cycle, from which policy decisions can be made for the time when cheap mineral phosphate (a fertiliser) is no longer available (in the next few decades) [2]. This example illustrates the complexity of the natural world, and how ‘grand’ is the challenge faced by biodiversity informatics to create a coordinated coupled modelling environment to address health, sustainability and environmental questions [78].

### How reliable are the data?

20. Science is, by its nature, a sceptical process. Data are received at face-value, examined and tested. If the user is satisfied, then the data will be applied. This process is crucial in biodiversity since information can rarely be generated by simple measurements. Concepts (like species), observations (based on human interpretations), proxy data (often originating from sensors) or algorithms (models fit for specific cases) constitute most biodiversity data with their inherent uncertainties and fuzziness. It is vital, then, that information about how the measurement was taken, to the minimum data standard, is included in the associated metadata. Judgement of quality involves an assessment of fitness-for-purpose and therefore cannot be an absolute measure. Data can of course contain both errors of fact, e.g. typographical errors, or errors of design, e.g. collecting data under a flawed methodology. Errors of fact can be detected by various means, e.g. duplicate entry or proof-reading whereas errors of design are more difficult to find automatically. A more significant problem is the accuracy of the data, meaning how precise and complete they are. In measurement it is accepted that a balance might weigh to the nearest 5 g, being a characteristic of the balance. In information terms, lacking a standard for generating the datum, it is harder. For instance, bibliographic citations can have diverse formats that humans can easily resolve to the same publication however computers, by and large, cannot unless given a PID as an information standard. The challenge for biodiversity informatics is to provide appropriate tools for data cleaning^m^ and to automate procedures for reading data for consistency [79], particularly against standard lists (see paragraph 16 above). Ultimately it is a case of *caveat emptor*. Users will develop trust in an information supplier and sites may wish to use a voting mechanism, e.g. similar to the supplier rating system on eBay. A system is required for data publishers to display comments from identifiable users (see paragraph 12 above), providing a feedback mechanism, essentially an open peer-review. Exposure to users is the best way to validate data.

### What will the physical infrastructure look like?

21. Plummeting cost of hardware, increasing use of virtualisation and blurring between fixed / mobile computing and work / domestic environments for computing makes the prediction of preferred computing environments of dubious value. Compiling this white paper has identified no apparent need for bespoke ICT technologies. A continued use of a wide variety of platforms and approaches is to be expected. Biodiversity informatics has many requirements in common with other informatics domains and it is noteworthy that biodiversity research, as in other disciplines has the potential to produce very large and rapidly growing data sets from, for example automated digitisation, remote sensing and genetic sequencing. Although the configuration of existing and planned cross-domain infrastructure such as LifeWatch supports biodiversity informatics well, the domain will place heavy capacity demands on the computing infrastructure in the medium-term. Hardware associated with sensor and data logging is addressed in Appendix 2. Like other domains, biodiversity informatics will require robustness, stability and persistence, so will likely rely on key institutions with long-term funding. Over the core hardware infrastructure lies a spatial data information infrastructure, the biodiversity component of which is largely the topic of this white paper. The leveraging of information from distinct but adjacent domains will be increasingly necessary in the future, such as digital literature resources, image, environmental and climatic information databases. As molecular methods find ever greater uptake, one particular set of resources will become increasingly important to biodiversity informatics: these are the many biomolecular resources that, within Europe, lie within the purview of the ELIXIR infrastructure [80]. While many of the core resources themselves may be sustained with comparatively long-term support, the services built upon these resources must be configured to include biodiversity science use cases. A unified voice in specifying these use cases is required from the biodiversity community. Building the ‘social infrastructure’, however, is a major challenge: we have the technological capability but we need to increase its uptake by the community. For that we need to strengthen considerably the socially connected network of experts spanning the two communities: ICT and biodiversity science.

## Section 3: new tools

### How much of the legacy collections can be digitised?

22. The world’s biological collections represent the hard core of biodiversity information. All other uses, from identification and naming onwards, are anchored in them. The collections contain an estimated 2–3 billion specimens but less than 10% have been catalogued in databases and much less captured as digital images [81,82]. This means that more than 90% of the collections are essentially unavailable for use through the Internet. Manually digitising collections represents an effort estimated at up to one million person years, but, with today’s mass-digitisation methodologies, the task is feasible. As shown by multiple virtual herbarium projects [83,84], the process can be partly automated through imaging techniques. With gazetteer services such as GeoLocate [85], georeferencing can also be computer-assisted. Another good example is the Volunteer site of the Atlas of Living Australia [86] whereby, when a backlog of digital images is available, their transcription is distributed through crowd-sourcing to a large number of volunteers. With help from initiatives like iDigBio [87], we envisage that distributed digitisation infrastructures will become essential parts of most major natural history collections and that dedicated services will be developed for outsourcing this task. A major challenge however is that collections still grow faster than they are being digitised (e.g. through endowments). As private collections must also be digitised by their owners, this requires a new suite of easy and inexpensive tools that can be deployed at large scale. To effectively deliver this research infrastructure service, digitisation requires prioritisation and its own funding channels.

### How to generate more targeted and reliable data?

23. Gathering information about the world around us has been a priority for biodiversity science for many years (see Appendix 3). Observatories will soon operate throughout the biosphere capturing different kinds of data over multiple scales, from microns to planet-wide, from parts of a second to years. It is very important to know the relative and also the absolute position of observed objects and events. This brings special challenges when observing the desired phenomenon and operating in extreme environments, such as the deep sea. The infrastructure for biodiversity data urgently needs more advanced informatics, support - not only mainstream ICT development but also the ability to deal with the specifics of biodiversity features and data^n^. It requires informatics to support observations, event detections, species identification, data transfer, storage, filtering and other kinds of data processing. New data-gathering tools that will allow new observatories at all biological scales and sensor networks covering the globe need to be designed, created and tested. There should be automatic processes allowing for feedback from data interpretation back to the observation or detection at site. This combination of techniques and related biodiversity informatics tools is expected to herald a revolution in biodiversity research, resolving much of our current fragmented data coverage and knowledge. Public-private partnerships should be encouraged to enter pre-competitive research and development in this evolution.

### What role do mobile devices play?

24. Developments in mobile communications offer numerous opportunities for innovation (see Appendix 2). Smartphones and tablet PCs with on-board GPS location can be easily taken into the field, creating opportunities both for innovative data collection and user information services. They are also particularly innovative for reference products such as identification keys^o^. Apps like these can be used to generate image-vouchered, location-tagged observations uploaded to central databases^p^. Performing science in large virtual communities, where participants have varied levels of expertise requires new techniques for data harvesting, processing, cleansing and validation.

### How do you find the data you need?

25. Most biodiversity data that now exists are semi-structured and can be searched with typical search tools (Google, GBIF, etc.). However, these are often designed for use by humans rather than for automated data retrieval tasks and may have in-built limitations or constraints. To make better use of general purpose tools, users may need to use more specialised resources as well. GBIF, for example, supports retrieval by species name but the user may also need to use resources such as the Catalogue of Life to provide alternative names for species-based searching. The volume of data now being searched is so large that it is often not possible to refine keyword-type searches sufficiently to recover the needle buried in the haystack, especially in the absence of widely-used vocabularies. Contextualising information (establishing relationships between data elements) in a resource is possible^q^, but currently difficult and slow. The implementation of PIDs (see Paragraph 7) would make the construction of metadata portals much easier. A mature search mechanism that contextualises rather than simply indexing would be far more powerful. A number of newly developed techniques exist, and some are under development, that make extensive use of visualisation methods to detect patterns and issues in data collections. These could be useful for quality and fitness-for-use assessment, especially in very large datasets such as the LTER-Europe data index or the GBIF index and taxonomical nomenclators. Data publishers need to go further in helping users find the data that match their requirements, with the use of PIDs, vocabularies and KOS (see paragraph 17).

### How do you extract the data you need?

26. In publications, either paper or PDF, information is often embedded in text blocks or tables in a way that inhibits its re-use. Semantic technologies (data mining) offer potential for liberating such data, but have not yet demonstrated the necessary flexibility or speed needed for broad uptake in the verbose, descriptive disciplines of taxonomy and ecology. Perversely, it is also difficult to extract information from highly condensed scientific writing such as taxonomic descriptions because this style of writing relies on implicit context in order to be understandable. New tools will be needed that use the vocabularies, ontologies and KOS described above to establish context between data elements, then to extract and assemble those elements into a format suitable for the user's purposes. Copyright held by commercial publishers remains a serious obstacle to recovering the non-copyright factual data. Some older publications that are not in copyright are being digitised, but serious issues remain over the rate at which the historic legacy literature is being captured, the completeness of the digitised literature and ease of access to this literature. Errors in the Optical Character Recognition (OCR) process mean that search of these archives will return an incomplete result set [88]. This presents an additional level of difficulty over and above the problems with extracting information from the born-digital literature.

### How do you aggregate the data you need?

27. For many analyses it is often necessary to aggregate data from several sources. Several data-aggregating initiatives have emerged in the last two decades for various areas of biodiversity informatics. Some of these initiatives were done on a project basis, while others were embedded in national structures, making them more reliable sources of information in the long-term. Examples include GBIF for primary data, Encyclopedia of Life (EOL) [89] for species descriptions, uBio [90] and Global Names Index [38] for names usages, CoL [40] for validated species names and Europeana [91] for multimedia resources. These data-aggregation initiatives have important beneficial side effects for biodiversity informatics such as enhancing the availability, standardisation and duplication (‘backup’) of data. Aggregation problems remain, similar for all these initiatives, such as hidden duplication, proper attribution, harvesting and storage. Each aggregrator has tended to solve these on their own by developing their own data provider network and internal infrastructure. Increasingly, though they are recognising the need to streamline and to avoid duplication of effort (for some examples see [18]). To facilitate integration, it will be valuable to develop a well-known catalogue of large-scale resources, with associated metadata, including a concept map.

### How complete are the data?

28. When combining data from different sources and domains, interoperability is clearly not the only obstacle to analysing complex patterns of biodiversity. The accessible biodiversity data today come largely from repositories and individual researchers, and are generally of high quality with respect to reliability. The quality is rather low however, with respect to consistency. In other words the data aggregated today has been collected for different purposes and on different spatio-temporal scales leaving significant gaps in the assembled data sets and seriously hampering the analysis of complex patterns with data from diverse domains. Gaps can be filled by developing more comprehensive biodiversity observatory networks (BONs) and associated e-tools to support the collection, aggregation, and discovery of data from these observatories. There is also a fundamental need to re-consider what we already have – to ‘invert the infrastructure’ [92],p.20] – to re-examine and re-formulate existing data to make it more homogeneous, to remove non-biodiversity factors (e.g. compensating for differing observation technique) and to make it suited to the kinds of analyses we foresee for the future.

### How can we encourage virtual research environments?

29. Virtual research environments (VREs), or Virtual Laboratories are online systems helping researchers to carry out their work. They include environments both to publish data (e.g. Scratchpads [93]) and to execute operations on data (e.g. myExperiment [94]) or both (e.g. AquaMaps [95] and iMarine [96]). VREs also include facilities to support collaboration between individuals. The challenge is to build integrative flexible e-Science environments using standardised building blocks and workflows, with access to data from various sources. Just as with physical laboratories, different kinds of VREs are possible, ranging from general-purpose to the highly specialised. A general VRE for wetland studies can be customised to a specific geographical area and populated with relevant datasets. A VRE specialised for a single scientific objective e.g. to find an optimal way of sequestering carbon in a forest would be equipped with workflows based on highly specialised simulation tools such as Biome-BGC [97]. For a successful uptake of VREs, they must generate immediate benefit for their users. For casual users the interface(s) must be simple and intuitive. For developers, there must be a usable pool of services and other resources that can be linked simply (e.g. BioVeL [98]). VREs must perform functions that people find useful. VREs as envisaged here, also act as social networking applications and have a central role in making some of the available technology described above usable or better, invisible to the majority of users.

### What can you do with your data in the future?

30. A biodiversity e-infrastructure should go much further than the generation, transfer, storage, and processing of biodiversity data. Applications demand that the infrastructure supports deploying the data in analysis, predictive modelling and decision support. The complexity of biodiversity includes systems interacting in chaotic and non-linear processes, extreme system effects and interactions between microscopic and macroscopic global levels, as well as on multiple time-scales. Understanding biodiversity is far more complex than understanding either meteorology or climate-change. To address problems that we cannot now handle we need:

• User friendly VREs with:

• interoperable and easily configurable components;

• access to real-time data (sensor, earth observation, weather, etc.);

• pre- and post-processing capabilities.

• Predictive multi-scale models;

• Feedback mechanisms to prompt new data generation (remote observations and measurements);

• Methods for integrated interaction between data, parameters, models and visualized results (“fine tuning” experiments, computational steering);

• New approaches for decision support when model outcomes result in various scenarios.

## Section 4: the human interface

### How can we give users confidence?

31. Experience suggests that for an effective outcome in biodiversity informatics, a balance of top-down and bottom-up approaches is required. It is also important to remember that there is small benefit to asking end-users for their requirements, when they may not be aware of the benefits that new technologies can bring. The FP5 funded project ENBI (European Network for Biodiversity Information, funded 2003–2005) [99] concluded that a modular infrastructure could provide both the architecture and the sustainability to overcome the partial and ad hoc solutions developed in the past twenty years, and designed the LifeWatch infrastructure for biodiversity and ecosystem research (an ESFRI project [100]). This approach, followed for a decade, has not led to the development of trusted repositories or stable funding, and therefore has not generated user confidence. Thus far, all biodiversity information projects share a common problem, viz. how to keep the service running after the funding period. If people don't have confidence that an environment will last essentially indefinitely, or at least as long as it is relevant to enter information, then why would they invest their time and effort contributing to the common system? Paper publication is perceived, unrealistically, to last indefinitely and is the yardstick by which people judge any new approach. Publishing in PDF format is conceptually equivalent to paper, although it’s easier, faster and cheaper to distribute copies and its longevity has not been demonstrated. New paradigms are making data available in forms that can be readily re-used, e.g. Pensoft Publishers [56] and GBIF's Integrated Publishing Toolkit [76]. It may be possible that international organisations, such as GBIF, or large national institutions, such as natural history museums, will agree to underwrite data services on a care-and-maintenance basis, while the underlying software goes open source but with institutional oversight. Ultimately, to build the crucial user confidence, service managers need to invest far more than they have thus far in marketing to create new social norms.

### Who owns what?

32. The traditional citation system provides a mechanism to measure impact and provenance but it applies to a publication unit rather than code from a software library or data from a repository. Systems are required that will generate comparable metrics from the new open science resources (see paragraph 15). Data contributors would benefit by knowing the number of people and/or projects who used those data (impact). Code developers will benefit by knowing how widely their code is being used (impact). Users want to be able to drill down and find who wrote the code or generated the data (provenance). The ability to credit the data creator or code author is the primary basis for trust in the quality of the data or service. The following challenges need to be resolved: first, we need a system of attribution that is robust in a distributed network, easily achieved by the use of PIDs and author identities (see paragraphs 7 and 11). Second, licensing is poorly understood in the community, both by producers and consumers. For data, the flavours of Creative Commons licenses that involve "non-commercial" clauses make risk-averse consumers wary of using material, even when free use was the intention of the original contributor. For software, the terms of open source licencing and free-use are similarly subtle. In both cases, there is a widespread failure to understand the distinction between licensing and copyright. Third, copyright often creates a barrier to data use and re-use, although in academic work no instances of case law have been identified, so guidance is based on commercial publishing case law, predicated on financial loss. The wider Open Science movement is pushing hard to clarify this situation and biodiversity data should benefit from the increasing widespread liberalisation.

### What benefits come to contributors?

33. Career progression is enormously influenced by citation metrics as a proxy for impact and, more than anything else, this keeps us tied to a paper publication model. Products that users want, e.g. identification keys, are often used without citation and contribute nothing to career progression. People are too often not sharing their data freely, but save it for their close collaborators: they need to be given new tools that facilitate data sharing in the long run, but keep them in control while the research is still active. New metrics need to be defined that measure how often a data set is used and where conclusions based (in part) on those data appear. This through-tracking requires, at the very least, two of the fundamentals discussed above in Section 1, the use of PIDs to track the data and the development of a system to identify contributors (= authors). Ultimately, this is the single largest problem we face in persuading people share their data.

## Endnotes

^a^ By a "coordinated coupled modelling environment" we mean a technological framework of interoperability that allows researchers to bring together different data and algorithms without undue difficulty for analysis, modelling and prediction. Such a framework could assist us to better understand biodiversity as a comprehensive, complex, integrated system rather than as an assemblage of species (or any other biological organisation). This comprehensive systems-oriented framework would be built from diverse but interlinked data and tools for data discovery and analysis across dimensions of scale of phenomena, time, space and disciplines (biology, chemistry, climatology, economics, sociology, geography). The effect of impacts and processes can then be assessed across temporal, spatial, and organisational (e.g. gene, individual, species, ecosystems) dimensions. For an alternative impression, refer to Virtual Physiological Human (VPH) for an analogous objective, as described by [101]:

“… a technological framework that aims to be descriptive, integrative and predictive.

### Descriptive

The framework should allow observations made in laboratories, in hospitals and in the field, at a variety of locations situated anywhere in the world, to be collected, catalogued, organized, shared and combined in any possible way.

### Integrative

The framework should enable experts to analyse these observations collaboratively, and develop systemic hypotheses that incorporate the knowledge of multiple scientific disciplines.

### Predictive

The framework should facilitate the interconnection of predictive models defined at different scales, with different methods and with different levels of detail, producing systemic networks that breathe life into systemic hypotheses; simultaneously, the framework should enable their validity to be verified by comparison with other clinical or laboratory observations.”

^b^ Based on the Lister definition of biodiversity, [102]: ‘Biodiversity is the variety, distinctiveness and complexity of all life on Earth, including its structures, functions, cultures, and information at all scales (from genetic to global) and in all its contexts (from DNA to self organization)’.

^c^ A valid name is the correct biological name of a taxon, determined according to the relevant rules of nomenclature.

^d^ At the International Conference on Research Infrastructures (ICRI2012), Copenhagen, 21–23 March 2012.

^e^ For a working definition of biodiversity informatics see http://en.wikipedia.org/wiki/Biodiversity_informatics

^f^ Related ‘future’ initiatives are presently being coordinated at the global level by the FP7 funded CReATIVE-B project (http://creative-b.eu/) and by GBIF (http://www.gbif.org/) through its Global Biodiversity Informatics Conference (Copenhagen, 2–4 July 2012) and subsequent Global Biodiversity Informatics Outlook publication (in preparation).

^g^ The EU vision for 2050 is: “Biodiversity and ecosystem services – the world’s natural capital – are preserved, valued and, insofar as possible, restored for their intrinsic value and so that they can continue to support economic prosperity and human well-being as well as avert catastrophic changes linked to biodiversity loss.” [http://ec.europa.eu/environment/nature/biodiversity/policy/].

^h^ The EU target for 2020 is to: “halt the loss of biodiversity and ecosystem services in the EU by 2020 and restore them insofar as possible, and step up the EU’s contribution to averting global biodiversity loss.” [http://ec.europa.eu/environment/nature/biodiversity/policy/].

^i^ BiSciCol project [http://biscicol.blogspot.co.uk/p/home.html] is one example of an attempt to do that.

^j^ At the non-European and global levels important projects include: DataONE, iDigBio, Atlas of Living Australia, Catalogue of Life, COOPEUS, CReATIVE-B, EOL, GBIF, GSC Biodiversity WG, TreeBase, CBOL and many more.

^k^ BioVeL in particular is a pilot implementation following the architecture and technical approach envisaged for the ESFRI LifeWatch Research Infrastructure for biodiversity science and ecosystems research.

^l^ A name usage is a statement that includes a name. The GNUB connects names with their usage in the literature, collections, etc.

^m^ See for example, how Atlas of Living Australia approaches this problem: http://www.ala.org.au/aboutthe-atlas/how-we-integrate-data/data-quality-assurance/.

^n^ The situation today can be likened to that which existed in the fields of meteorology and climatology in the 1960’s and 70’s when the emergence of numerical weather prediction drove the demand for new observations and the emergence of a global infrastructure for acquiring data.

^o^ The EC KeyToNature project (http://www.keytonature.eu) developed a series of apps for identifying species in the field.

^p^ For example Artportalen in Sweden (http://www.artportalen.se/default.asp), Ornitho in Italy (http://www.ornitho.it/) and Project Noah in the USA (http://www.projectnoah.org/).

^q^ For example sig.ma (http://sig.ma/).

### Appendix 1

A moderated mailing list has been established for the Biodiversity Informatics Community. To join the list, e-mail DR (dmr@nomencurator.org). Contributing authors to this White Paper are, in alphabetical order:

Wouter Addink, ETI Bioinformatics, NL

Bart Aelterman, Research Institute for Nature and Forest (INBO), BE

Donat Agosti, Plazi, CH

Linda Amaral-Zettler, Marine Biological Laboratory, US

Arturo H. Ariño, Universidad de Navarra, ES

Christos Arvanitidis, Hellenic Center for Marine Research, GR

Thierry Backeljau, Royal Belgian Institute for Natural Sciences, BE

Nicolas Bailly, WorldFish Center, PH

Lee Belbin, Atlas of Living Australia, AU

Walter Berendsohn, Botanischer Garten und Botanisches Museum Berlin-Dahlem, Freie Universität Berlin, DE

Nic Bertrand, Centre for Ecology and Hydrology, Lancaster, UK

Neil Caithness, Oxford University, UK

David Campbell, The Paleontological Research Institution, US

Guy Cochrane, EMBL - European Bioinformatics Institute Hinxton, UK

Noël Conruyt, Université de la Réunion, FR

Alastair Culham, University of Reading, UK

Christian Damgaard, Aarhus University, DK

Neil Davies, UC Berkeley, US

Bruno Fady, INRA, UR629 Ecologie des Forêts Méditerranéennes (URFM) and CESAB (CEntre de Synthèse et d'Analyse sur la Biodiversité), FR

Sarah Faulwetter, Hellenic Center for Marine Research, GR

Alan Feest, Bristol University, UK

Dawn Field, Oxford University, UK

Eric Garnier, UMR5175 Centre d'Ecologie Fonctionnelle & Evolutive and CESAB (CEntre de Synthèse et d'Analyse sur la Biodiversité), FR

Guntram Geser, Salzburg Research Forschungsgesellschaft, AT

Jack Gilbert, University of Chicago, US

Bernd Grosche, Federal Office for Radiation Protection, DE

David Grosser, Université de la Réunion, FR

Alex Hardisty, Cardiff University, UK

Bénédicte Herbinet, Fondation pour la Recherche sur la Biodiversité, FR

Donald Hobern, GBIF Secretariat, DK

Andrew Jones, Cardiff University, UK

Yde de Jong, Universiteit van Amsterdam, NL

David King, The Open University, UK

Sandra Knapp, Natural History Museum, London, UK

Hanna Koivula, Finnish Museum of Natural History, FI

Wouter Los, University of Amsterdam, NL

Chris Meyer, Smithsonian Institution, US

Robert A. Morris, UMASS-Boston and Harvard University Herbaria, US

Norman Morrison, University of Manchester, UK

David Morse, The Open University, UK

Matthias Obst, University of Gothenburg, SE

Evagelos Pafilis, Hellenic Center for Marine Research, GR

Larry M. Page, Florida Museum of Natural History, US

Roderic Page, University of Glasgow, UK

Thomas Pape, Natural History Museum of Denmark, DK

Cynthia Parr, Smithsonian Institution, US

Alan Paton, Royal Botanic Gardens, Kew, UK

David Patterson, Marine Biological Laboratory, Woods Hole, US

Elisabeth Paymal, Fondation pour la Recherche sur la Biodiversité, FR

Lyubomir Penev, Pensoft Publishers, BG

Marc Pollet, Research Institute for Nature and Forest (INBO), BE

Richard Pyle, Bishop Museum, Honolulu, US

Eckhard von Raab-Straube, Botanischer Garten und Botanisches Museum Berlin-Dahlem, Freie Universität Berlin, DE

Vincent Robert, Centraalbureau voor Schimmelcultures, NL

Dave Roberts, Natural History Museum, London, UK

Tim Robertson, GBIF Secretariat, DK

Olivier Rovellotti, Natural Solutions, FR

Hannu Saarenmaa, Finnish Museum of Natural History, FI

Peter Schalk, ETI Bioinformatics, NL

Joop Schaminee, Wageningen UR and Radboud University Nijmegen, NL

Paul Schofield, University of Cambridge, UK

Andy Sier, Centre for Ecology & Hydrology, UK

Soraya Sierra, Stichting Naturalis Biodiversity Center, NL

Vince Smith, Natural History Museum, London, UK

Edwin van Spronsen, ETI Bioinformatics, NL

Simon Thornton-Wood, University of Reading, UK

Peter van Tienderen, Universiteit van Amsterdam, NL

Jan van Tol, Stichting Naturalis Biodiversity Center, NL

Éamonn Ó Tuama, GBIF Secretariat, DK

Peter Uetz, Virginia Commonwealth University, US

Lea Vaas, Centraalbureau voor Schimmelcultures, NL

Régine Vignes Lebbe, University Pierre et Marie Curie, FR

Todd Vision, University of North Carolina at Chapel Hill, US

Duong Vu, Centraalbureau voor Schimmelcultures, NL

Aaike De Wever, Royal Belgian Institute for Natural Sciences, BE

Richard White, Cardiff University, UK

Kathy Willis, University of Oxford, UK

Fiona Young, University of Reading, UK

### Appendix 2

#### Mobilising economic benefits

At present, 87% of the world’s population have mobile phone subscriptions and 1.2 billion of these are mobile Web users. In 2011, almost half a billion smartphones were shipped globally, exceeding sales of PCs [103]. In 2010, 300,000+ Smartphone apps were downloaded 10.9 billion times. Prediction is that in 2014 some 77 billion apps will be downloaded representing an estimated US$35 billion market [104]. With the 10 times faster 4G mobile networks as successor of 3G already available in some countries, high speed bandwidth to mobile devices will facilitate online use of services demanding bandwidth such as video-streaming.

Next generation apps, incorporating stable content, smart algorithms and location-awareness in combination with multiple layers of online data delivered over 4G bandwidth (not yet available in Europe), offer the promise of highly innovative information products that can serve markets in both the science and social domains, provided the data are made available to serve these needs.

The EC KeyToNature project [105] developed a series of apps for identifying species in the field demonstrating that there is a market for quality taxonomic reference works that can contribute to cost recovery. This approach however does not come without risk. The mobile devices’ field is evolving extremely fast and apps developed for a device are out of business only one or two years later.

## Appendix 3

### Gathering biodiversity data

Gathering biodiversity data can be divided into 3 main routes:

### *Remote sensing*

Earth observation at multiple wavelengths by aeroplane, satellite and ground-based sensors are in the early stages of development for biodiversity observation. They are largely based on surveillance technologies and require the development of new techniques to process the type of data they produce, both in routine monitoring and the detection of rare events. New generations of sensors designed for biodiversity observation are needed. Camera traps today and DNA chip sensors tomorrow could transmit data wirelessly, and be linked directly to researcher’s desks. Even with existing technology, it is becoming economically feasible to collect large amounts of environmental data automatically. This approach will undoubtedly present a significant new challenge in handling very large data volumes [106].

### *Environmental metagenomics*

"Grind and find" techniques allows the study of many organisms in a sample at the same time, presenting the challenge of scaling biodiversity observation from the molecule to the planet [107]. For example in November 2011, the Beijing Genomics Institute (BGI) launched its “Three Million Genomes Project”, an ambitious effort consisting of three sub-projects: “Million Plant and Animal Genomes Project”, “Million Human Genomes Project” and “Million Micro-Ecosystem Project”. In the latter, genomes of more than 600 microbial species, including over 3,500 strains and 1,800 metagenomes have already been completed. Projects like these are generating in the order of 20 petabytes of data per year. With the unlimited influx of sequence data being a real possibility, archives operating under the INSDC (International Nucleotide Sequence Database Collaboration) face a new situation in which it is no longer possible to archive all components of all datasets. Indeed, a community discussion is underway around decision-making that is informed by scientific and economic arguments about the aggressiveness to which different classes of sequence data should be compressed [108]. Given the value of samples from temporal environmental genomic studies with time-point specific elements (e.g. Ocean Sampling Day 2014 http://www.microb3.eu/work-packages/wp2) and limited or no opportunity to resample, contributions to the sequence compression debate from the biodiversity informatics community are essential.

### *Human observation*

Informatics should empower the human observer in the field and in the laboratory, improving observational data quality and providing for data transfer with automatic feedback mechanisms. Laboratory-based studies are increasingly being supported by electronic tools that are replacing the traditional paper laboratory notebook and increasingly, instruments are producing data feeds that can be directly integrated. It is often necessary to prepare baseline sample information that is used to interpret field information, for example use micro-CT scanning [109] to reveal details of three-dimensional structure. In field sites the infrastructure is either based on long term monitoring of selected parameters, or consists of small experimental plots where the response of controlled biodiversity systems on parameter change can be detected. Examples of the latter are mesocosms or plant communities in laboratory conditions. Long term monitoring is quite well developed in the LTER-Europe network (Long term Ecological Research monitoring), the MARS network of marine stations, GLEON (Global Lake Ecological Observatory Network) [110], NEON (National Ecological Observatory Network) [111] and the Swedish Taxonomy Initiative [112]. These monitoring networks produce vast amounts of biodiversity data and a common data infrastructure is yet only developed for the metadata.

### Author’s contributions and acknowledgements

This article was complied from contributions to a public GoogleDoc (http://is.gd/WhitePaperChapters) and notice of the consultation was widely circulated through mailing lists and presentation at conferences. Contributions were either additions to the text or in the form of comments, some by e-mail. Continuity editing was performed jointly by AH and DR. The fundamental structuring of the article has been determined by AH, NM, DR, HS and PvT. The unnumbered sections (Grand Challenge, Recommendations, Preface, Context, Taxonomic Impediment, Changing the landscape - A decadal vision, Realising the vision) have been written by AH and DR. Many individuals have helped with the overall creation of this article, either by providing contributions and comments on the sections previously referred to or by contributing content for the numbered paragraphs 1 – 33. This has been by direct contribution of text or as the source of material on specific points or by providing comments and points of clarification.

We thank each one of them. The editing authors would also like to extend particular thanks to Niobe Haitas for her invaluable help for proof-reading and editing some late drafts of this article.

The work reported in this article forms part of the coordination and support activities (dissemination, outreach, community building) being carried out by the BioVeL and ViBRANT projects. BioVeL is funded by the European Union 7th Framework Programme within the Research Infrastructures group, grant no. 283359. ViBRANT is funded by the European Union 7th Framework Programme within the Research Infrastructures group, grant no. 261532.

### Hardisty, Roberts & biodiversity informatics community

See Appendix 1 for a complete list of the 80 contributors and their affiliations.

## References

[B1] Hodge A Feed the world? arbuscular mycorrhiza and agriculture Microbiology Today 2012 39 90 93 http://www.sgm.ac.uk/download.cfm/docid/83F7DB84-8FB3-4801-BD5A83582EB91F70

[B2] Gilbert N Environment: the disappearing nutrient Nature News 2009 461 716 718 10.1038/461716a 19812648

[B3] Purves D Scharlemann JPW Harfoot M Newbold T Tittensor DP Hutton J Emmott S Ecosystems: time to model all life on earth Nature 2013 493 295 297 23325192 10.1038/493295a

[B4] The Royal Society Science as an open enterprise. Final report June 2012 http://royalsociety.org/uploadedFiles/Royal_Society_Content/policy/projects/sape/2012-06-20-SAOE.pdf

[B5] Davies N Field D The Genomic Observatories Network Sequencing data: a genomic network to monitor earth Nature 2012 481 145 doi:10.1038/481145a 22237100 10.1038/481145a

[B6] Davies N Meyer C Gilbert JA Amarel-Zettler L Deck J Bicak M Rocca-Serra P Assunta-Sansone S Willis K Field D A call for an international network of genomic observatories (GOs) GigaScience 2012 1 5 doi:10.1186/2047-217X-1-5 23587188 10.1186/2047-217X-1-5 PMC3617453

[B7] He F Hubbell SP Species-area relationships always overestimate extinction rate from habitat loss Nature 2011 473 368 371 http://dx.doi.org/10.1038/nature09985 10.1038/nature09985 21593870

[B8] Knight R Biodiversity loss: how accurate are the numbers? 2012 The BBC News Magazine http://www.bbc.co.uk/news/magazine-17826898

[B9] Palmer MA Febria CM The heartbeat of ecosystems Science 2012 336 6087 1393 1394 http://dx.doi.org/10.1126/science.1223250 10.1126/science.1223250 22700910

[B10] Relman DA Learning about who we are Nature 2012 486 194 195 http://dx.doi.org/10.1038/486194a 10.1038/486194a 22699602

[B11] Kau AL Ahern PP Griffin NW Goodman AL Gordon JI Human nutrition, the gut microbiome and the immune system Nature 2011 474 327 336 10.1038/nature10213 21677749 PMC3298082

[B12] Christensen NL Bartuska AM Brown JH Carpenter S D’Antonio C Francis R Franklin JF MacMahon JA Noss RF The report of the ecological society of America committee on the scientific basis for ecosystem management Ecol Appl 1996 6 665 691 10.2307/2269460

[B13] Europa press releases http://europa.eu/rapid/pressReleasesAction.do?reference=SPEECH/12/258

[B14] Nagoya protocol on access to genetic resources and the fair and equitable sharing of benefits arising from their utilization to the Convention on Biological Diversity http://treaties.un.org/doc/source/signature/2010/CN782E.pdf

[B15] Convention on Biological Diversity tenth meeting of the Conference of the Parties (COP 10) http://www.cbd.int/cop10/?section=welcome

[B16] Hardisty AR Peirce SC Preece A Bolton CE Conley EC Gray WA Rana OF Yousef Z Elwyn G Bridging two translation gaps: a new informatics research agenda for telemonitoring of chronic disease Int J Med Inform 2011 80 734 744 http://dx.doi.org/10.1016/j.ijmedinf.2011.07.002 10.1016/j.ijmedinf.2011.07.002 21890403

[B17] Peterson AT Knapp S Guralnick R Soberón J Holder MT The big questions for biodiversity informatics Syst Biodivers 2010 8 159 168 http://dx.doi.org/10.1080/14772001003739369 10.1080/14772001003739369

[B18] Parr CS Guralnick R Cellinese N Page RDM Evolutionary informatics: unifying knowledge about the diversity of life Trends Evol Ecol 2012 27 94 103 http://dx.doi.org/10.1016/j.tree.2011.11.001 10.1016/j.tree.2011.11.001 22154516

[B19] Dean J Ghemawat S MapReduce: simplified data processing on large clusters Proceedings of the 6th symposium on operating systems design and implementation (OSDI '04): December 6–8 2004 2004 San Francisco, California: USENIX: The Advanced Computing Systems Association 379 394

[B20] De Meester L Van Tienderen P Werger M Hector A Wörheide G Niemelä J Aguilar A Smets E Godfray C Sutherland W Bauhus J Courchamp F Gandini G Kochn M Le Maho Y Manuel M Pawlowski J Quéinnec E Owens I Keustermans L The LERU Biodiversity Working Group: Challenges for biodiversity research in Europe Procedia Social and Behavioral Sciences 2011 13 83 100 10.1016/j.sbspro.2011.03.007

[B21] GEO BON Principles of the GEO BON information architecture (version 1.0) http://www.earthobservations.org/documents/cop/bi_geobon/geobon_information_architecture_principles.pdf

[B22] GEO BON Implementation overview 2008 http://www.earthobservations.org/documents/geo_v/07_%20GEO%20Bon%20-%20Implementation%20Overview.pdf

[B23] GEO BON Detailed implementation plan (version 1.0) 2010 http://earthobservations.org/documents/cop/bi_geobon/geobon_detailed_imp_plan.pdf

[B24] The Global Earth Observation System of Systemshttp://www.earthobservations.org/geoss.shtml

[B25] e-science European infrastructure for biodiversity and ecosystem researchhttp://www.lifewatch.eu/

[B26] European Biodiversity Observation Network http://www.ebone.wur.nl/UK/

[B27] European Biodiversity Observation Networkhttp://www.eubon.eu

[B28] Convention on Biological Diversity – taxonomic impedimenthttp://www.cbd.int/gti/problem.shtml23593188

[B29] MacLeod N Automated taxon identification in systematics - theory, approaches and applications 2007 CRC Press

[B30] Swedish Species Gatewayhttp://www.slu.se/en/collaborative-centres-and-projects/swedish-lifewatch/news-archive/2011/9/betaversion/

[B31] Barcode of Lifehttp://www.barcodeoflife.org/

[B32] Moorea Biocode Projecthttp://mooreabiocode.org/

[B33] Costello MJ May RM Stork NE Can We name Earth's species before they Go extinct? Science 2013 339 6118 413 416 10.1126/science.1230318 23349283

[B34] Hampton SE Parker JN Collaboration and productivity in scientific synthesis BioScience 2011 61 900 910 http://dx.doi.org/10.1525/bio.2011.61.11.9 10.1525/bio.2011.61.11.9

[B35] Biodiversity information projects of the worldhttp://www.tdwg.org/biodiv-projects/projects-database

[B36] Patterson DJ Cooper J Kirk PM Pyle RL Remsen DP Names are key to the big new biology Trends Evol Ecol 2010 25 686 691 doi:10.1016/j.tree.2010.09.004 10.1016/j.tree.2010.09.004 20961649

[B37] Global Names Architecturehttp://globalnames.org/

[B38] Global Names Indexhttp://gni.globalnames.org/

[B39] Global Names Usage Bank http://www.globalnames.org/GNUB/

[B40] Species2000 / ITIS catalogue of lifehttp://www.catalogueoflife.org/

[B41] NCBI taxonomy databasehttp://ncbi.nlm.nih.gov/taxonomy/

[B42] WOrld register of Marine Specieshttp://www.marinespecies.org/

[B43] iPlant Taxonomic Name Resolution Servicehttp://tnrs.iplantcollaborative.org/

[B44] Zoobank: the official registry of zoological nomenclaturehttp://zoobank.org/

[B45] Indexing for lifehttp://www.i4life.eu/

[B46] Alroy J How many named species are valid? P Natl Acad Sci USA 2002 99 3706 3711 http://dx.doi.org/10.1073/pnas.062691099 10.1073/pnas.062691099 PMC122588 11891342

[B47] Mora C Tittensor DP Adl S Simpson AGB Worm B How many species Are there on earth and in the ocean? PLoS Biol 2011 9 8 e1001127 doi:10.1371/journal.pbio.1001127 10.1371/journal.pbio.1001127 21886479 PMC3160336

[B48] Schindel DE Miller SE Polaszek A Provisional nomenclature: the on-ramp to taxonomic names Systema naturae 250 - The Linnaean Ark 2010 CRC Press 109 116 http://dx.doi.org/10.1201/EBK1420095012-c10

[B49] DataCitehttp://www.datacite.org/

[B50] Linked Datahttp://linkeddata.org/

[B51] Constable H Guralnick R Wieczorek J Spencer C Peterson AT VertNet: a new model for biodiversity data sharing PLoS Biol 2010 8 2 http://dx.doi.org/10.1371/journal.pbio.1000309e1000309 10.1371/journal.pbio.1000309 PMC2821892 20169109

[B52] Bonney R Cooper CB Dickinson J Kelling S Phillips T Rosenberg KV Shirk J Citizen science: a developing tool for expanding science knowledge and scientific literacy BioScience 2009 59 977 984 http://dx.doi.org/10.1525/bio.2009.59.11.9 10.1525/bio.2009.59.11.9

[B53] Delaney DG Sperling CD Adams CS Leung B Marine invasive species: validation of citizen science and implications for national monitoring networks Biol Invasions 2008 10 117 128 http://dx.doi.org/10.1007/s10530-007-9114-0 10.1007/s10530-007-9114-0

[B54] TinEye Reverse Image Searchhttp://www.tineye.com/

[B55] Arvanitidis C Faulwetter S Chatzigeorgiou G Penev L Bánki O Dailianis T Pafilis E Kouratoras M Chatzinikolaou E Fanini L Vasileiadou A Pavloudi C Vavilis P Koulouri P Dounas C Engaging the broader community in biodiversity research: the concept of the COMBER pilot project for divers in ViBRANT Zookeys 2011 150 211 229 10.3897/zookeys.150.2149 22207815 PMC3234440

[B56] Semantic markup and publishing. Pensoft Publishers http://www.pensoft.net/page.php?P=14

[B57] ORCIDhttp://about.orcid.org/

[B58] Virtual International Authority Filehttp://www.oclc.org/viaf/default.htm

[B59] Piwowar H Altmetrics: value all research products Nature 2013 493 159 159 23302843 10.1038/493159a

[B60] Kuipers T Van der Hoeven J Insight into digital preservation of research output in Europe. PARSE. Insight Project deliverable D3.4 Survey Report 2009 http://www.parse-insight.eu/downloads/PARSE-Insight_D3-4_SurveyReport_final_hq.pdf

[B61] Science Staff Introduction to special issue dealing with data Science 2011 331 692 693 http://dx.doi.org/10.1126/science.331.6018.692 21311002 10.1126/science.331.6018.692

[B62] Tenopir C Allard S Douglass K Aydinoglu AU Wu L Read E Manoff M Frame M Data sharing by scientists: practices and perceptions PLoS One 6 6 e21101 http://dx.doi.org/10.1371/journal.pone.0021101 21738610 10.1371/journal.pone.0021101 PMC3126798

[B63] Williams R Pryor G Bruce A Macdonald S Marsden W Calvert J Dozier M Neilson C Patterns of information use and exchange: case studies of researchers in the life sciences Research Information Network 2009 http://rin.ac.uk/system/files/attachments/Patterns_information_use-REPORT_Nov09.pdf

[B64] Leonelli S, Bastow R Making data accessible to all - GARNet and EGENIS workshop report: 12–13 July 2012 2012 Exeter, UK http://www.garnetcommunity.org.uk/sites/default/files/Making%20Data%20Accessibile%20to%20All%20Report%202012.pdf

[B65] OECD OECD principles and guidelines for access to research data from public funding 2007 Paris http://www.oecd.org/dataoecd/9/61/38500813.pdf

[B66] Gruber T Liu L, Tamer Ozsu M Ontology The encyclopedia of database systems 2009 Springer http://tomgruber.org/writing/ontology-definition-2007.htm

[B67] Singhal A Introducing the knowledge graph: things, not strings GoogleBlog 2012 http://googleblog.blogspot.co.uk/2012/05/introducing-knowledge-graph-things-not.html

[B68] Unified Medical Language System (UMLS) http://www.nlm.nih.gov/research/umls

[B69] Agrovoc Thesarushttp://aims.fao.org/standards/agrovoc/about

[B70] The Open Biological and Biomedical Ontologieshttp://obofoundry.org/

[B71] Environment Ontology Environment Ontologyhttp://www.environmentontology.com/

[B72] DAML Ontology Libraryhttp://www.daml.org/ontologies/

[B73] Wieczorek J Bloom D Guralnick R Blum S Döring M Darwin Core: an evolving community-developed biodiversity data standard PLoS One 2012 7 1 e29715 http://dx.doi.org/10.1371/journal.pone.0029715 10.1371/journal.pone.0029715 22238640 PMC3253084

[B74] Cryer P Hyam R Miller C Nicolson N Cryer P Hyam R Miller C Nicolson N Tuama EO Page R Rees J Riccardi G Richards K White R Adoption of persistent identifiers for biodiversity informatics Report published by GBIF Secretariat 2009 http://imsgbif.gbif.org/CMS_ORC/?doc_id=2956&download=1

[B75] Catapano T Hobern D Lapp H Morris RA Morrison N Noy N Schildhauer M Thau D Recommendations for the Use of knowledge organisation systems by GBIF Report published by GBIF Secretariat 2011 http://imsgbif.gbif.org/CMS_ORC/?doc_id=2942&download=1

[B76] GBIF The Integrated Publishing Toolkithttp://www.gbif.org/informatics/infrastructure/publishing

[B77] Friend F Guédon JC Van de Sompel H Beyond sharing and re-using: toward global data networking. GRDI2020 Project 2011 http://www.grdi2020.eu/pdf/Toward_Global_Data_Networking.pdf

[B78] Adam D Brown N Position statement on food security and safety 2011 UK: Report published by the Society for General Microbiology, Reading http://www.sgm.ac.uk/PA_Forms/FoodPS_Web.pdf

[B79] Mathew C Güntsch A A workflow for data refinement. BioVeL project newsletter No. 2, Autumn 2012 http://www.biovel.eu/images/publications/newsno2autumn12.pdf

[B80] ELIXIR infrastructure for biological informationhttp://www.elixir-europe.org/

[B81] Ariño AH Approaches to estimating the universe of natural history collections data Biodiversity Informatics 2010 7 81 92

[B82] Duckworth WD Genoways HH Rose CL Preserving natural science collections: chronicle of our environmental heritage 1993 Washington, DC: National Institute for the Conservation of Cultural Property

[B83] Virtual herbarium at the Botanic Garden And Botanical Museum Berlin-Dahlem http://ww2.bgbm.org/Herbarium/default.cfm

[B84] Paris Natural History collectionshttp://coldb.mnhn.fr/

[B85] GEOLocate – a platform for georeferencing natural history collections datahttp://www.museum.tulane.edu/geolocate/22992364

[B86] Atlas of Living Australia Biodiversity Volunteer Portalhttp://volunteer.ala.org.au/

[B87] iDigBio Integrated Digitized Biocollectionshttps://www.idigbio.org/home

[B88] Wei Q Heidorn PB Freeland C Name matters: taxonomic name recognition (TNR) in biodiversity heritage library (BHL) iConference 2010 Proceedings 2010 284 288 http://hdl.handle.net/2142/14919

[B89] Encyclopedia of Lifehttp://eol.org/

[B90] Universal Biological Indexer and Organizerhttp://www.ubio.org/

[B91] Europeanahttp://www.europeana.eu/portal/

[B92] Edwards PN A vast machine: computer models, climate data, and the politics of global warming 2010 MIT Press

[B93] Scratchpads: biodiversity onlinehttp://scratchpads.eu/

[B94] myExperimenthttp://www.myexperiment.org/

[B95] Aquamapshttp://www.aquamaps.org/

[B96] iMarinehttp://www.i-marine.eu/

[B97] Biome-BGChttp://www.ntsg.umt.edu/project/biome-bgc/

[B98] Biodiversity Virtual e-Laboratoryhttp://www.biovel.eu/

[B99] European Network for Biodiversity Informationhttp://www.enbi.info

[B100] European Strategy Forum on Research Infrastructureshttp://ec.europa.eu/research/infrastructures/index_en.cfm?pg=esfri

[B101] Fenner JW Brook B Clapworthy G The EuroPhysiome. STEP and a roadmap for the virtual physiological human Phil. Trans. R. Soc. A 2008 366 1878 2979 2999 10.1098/rsta.2008.0089 18559316

[B102] Lister NM A systems approach to biodiversity conservation planning Environ Monit Assess 1998 49 123 155 10.1023/A:1005861618009

[B103] mobiThinkinghttp://www.mobithinking.com/

[B104] ABI researchhttp://www.abiresearch.com/

[B105] Key To Naturehttp://www.keytonature.eu

[B106] Nature Editorial NEON and on Nature 2011 476 125 http://dx.doi.org/10.1038/476125b 21833047 10.1038/476125b

[B107] Jones MB Schildhauer MP Reichman OJ Bowers S The New bioinformatics: integrating ecological data from the gene to the biosphere Ecol Evol Syst 2006 37 519 544 http://dx.doi.org/10.1146/annurev.ecolsys.37.091305.110031 10.1146/annurev.ecolsys.37.091305.110031

[B108] Cochrane G Cook CE Birney E The future of DNA sequence archiving GigaScience 2012 1 2 http://dx.doi.org/10.1186/2047-217X-1-2 23587147 10.1186/2047-217X-1-2 PMC3617450

[B109] Marbigenhttp://www.marbigen.org/content/microtomography

[B110] Global Lake Ecological Observatory Networkhttp://www.gleon.org

[B111] National Ecological Observatory Networkhttp://www.neoninc.org

[B112] ArtDatabanken Swedish species information centrehttp://www.slu.se/en/collaborative-centres-and-projects/artdatabanken

